# Re-examining Correlations Between Synonymous Codon Usage and Protein Bond Angles in *Escherichia coli*

**DOI:** 10.1093/gbe/evae080

**Published:** 2024-04-15

**Authors:** Opetunde J Akeju, Alexander L Cope

**Affiliations:** New Jersey City University, Jersey City, New Jersey, USA; Department of Genetics, Rutgers University, Piscataway, New Jersey, USA; Human Genetics Institute of New Jersey, Rutgers University, Piscataway, New Jersey, USA; Robert Wood Johnson Medical School, Rutgers University, Piscataway, New Jersey, USA

**Keywords:** codon usage, protein structure, population genetics

## Abstract

Rosenberg AA, Marx A, Bronstein AM (Codon-specific Ramachandran plots show amino acid backbone conformation depends on identity of the translated codon. Nat Commun. 2022:13:2815) recently found a surprising correlation between synonymous codon usage and the dihedral bond angles of the resulting amino acid. However, their analysis did not account for the strongest known correlate of codon usage: gene expression. We re-examined the relationship between bond angles and codon usage by applying the approach of Rosenberg et al. to simulated protein-coding sequences that (i) have random codon usage, (ii) codon usage determined by mutation biases, and (iii) maintain the general relationship between codon usage and gene expression via the assumption of selection-mutation-drift equilibrium. We observed correlations between dihedral bond angle and codon usage when codon usage is entirely random, indicating possible conflation of noise with differences in bond angle distributions between synonymous codons. More relevant to the general analysis of codon usage patterns, we found surprisingly good agreement between the analysis of the real sequences and the analysis of sequences simulated assuming selection-mutation-drift equilibrium, with 91% of significant synonymous codon pairs detected in the former were also detected in the latter. We believe the correlation between codon usage and dihedral bond angles resulted from the variation in codon usage across genes due to the interplay between mutation bias, natural selection for translation efficiency, and gene expression, further underscoring these factors must be controlled for when looking for novel patterns related to codon usage.

**Significance**

Synonymous mutations—those that alter the DNA but not the amino acid sequence of a gene—are hypothesized to impact protein folding, with a recent paper finding differences in the protein bond angle distributions of an amino acid are dependent on the synonymous codon used to encode the amino acid in the DNA. We re-examined this relationship using simulated protein-coding sequences, finding evidence that the apparent relationship between codon usage and protein bond angles is confounded by the relationship between codon usage and gene expression. Our findings further emphasize the need to consider differences in gene expression when testing for possible relationships between a molecular feature or property and codon usage.

## Introduction

Codon usage bias (CUB), or the non-uniform usage of synonymous codons, has been observed across all domains of life (Plotkin and Kudla 2011). CUB is driven by a combination of both non-adaptive (e.g. mutation biases) and adaptive (e.g. natural selection for translation efficiency/accuracy) evolutionary forces (Ikemura 1981; Gouy and Gautier 1982; Bulmer 1991; Drummond and Wilke 2008; Shah and Gilchrist 2011). Empirical work has shown that changes to synonymous codon usage can affect co-translational protein folding, with various computational studies seeking to determine if there is a general connection between codon usage and protein structure (Thanaraj and Argos 1996; Saunders and Deane 2010; Chaney 2017; Jacobs and Shakhnovich 2017; Cope and Gilchrist 2022). Rosenberg et al. (2022) recently explored the relationship between synonymous codon usage and the dihedral bond angles—*ϕ* and *ψ* angles of C*α*–N bond and the C*α*–C=O bond, respectively—that form a protein’s backbone. Applying a method they developed to compare dihedral bond angle distributions across synonymous codons to *E. coli* protein-coding sequences, they detected numerous statistically significant differences between the dihedral bond angle distributions of synonymous codons. Although they qualify their results by stating that correlation does not imply causation, they hypothesize that differences in dihedral bond angle distributions between synonymous codons could be due to differences in elongation speeds between codons. This hypothesis is supported by a correlation between the differences in the dihedral bond angles and the elongation speeds of the codons.

Gene expression is an important factor shaping the evolution of a protein on both micro and macroevolutionary timescales. For example, gene expression is correlated with the evolutionary rate—as measured via the ratio of nonsynonymous to synonymous substitutions—of proteins across all major domains of life (Pál et al. 2001; Drummond et al. 2006; Drummond and Wilke 2008; Zhang and Yang 2015). Numerous studies concluded that failure to control for differences in gene expression caused spurious correlations when quantifying protein evolution For example, while some studies concluded gene dispensability (Hirsh and Fraser 2001) and properties of the protein–protein interaction network (Fraser et al. 2002; Fraser 2005) correlated with evolutionary rate, other studies argued these correlations arose simply due to underlying correlations with gene expression (Bloom and Adami 2003, 2004; Pál et al. 2003; Batada 2007; Wang and Zhang 2009).

Gene expression is the strongest known correlate with CUB, with highly expressed genes biased towards codons that are translated more efficiently or accurately and lowly expressed genes biased towards codons favored by mutational biases (Drummond and Wilke 2008; Shah and Gilchrist 2011). A common population genetics model for studying codon usage patterns posits that codon usage is at selection-mutation-drift equilibrium (SMDE) (Li 1987; Bulmer 1991), and has been extended over the past decade to explicitly model the effects of gene expression on the strength of natural selection on codon usage (Shah and Gilchrist 2011; Wallace et al. 2013; Gilchrist et al. 2015). Although the work of Rosenberg et al. (2022) does not explicitly make statements about the nature of the evolution of CUB, the SMDE framework known as the Ribosomal Overhead Cost version of the Stochastic Evolutionary Model of Protein Production Rates (ROC-SEMPPR) provides an excellent tool for testing if the observations of Rosenberg et al. (2022) may be explained by the relationship between codon usage and gene expression. Previous work showed that failure to control for gene expression and other biases known to impact codon usage patterns (e.g., amino acid biases) led to spurious differences in the codon usage biases of signal peptides (Cope et al. 2018) and protein secondary structure (Cope and Gilchrist 2022). Along the same lines, signals of codon autocorrelation—the tendency for the same synonymous codon to be used at nearby amino acids within a gene more frequently than expected by chance (Cannarrozzi 2010)—could be explained simply by differences in codon usage across genes due to gene expression (Hussmann and Press 2014; Novoa et al. 2019). We present results using simulated data suggesting the specific findings of Rosenberg et al. (2022) are likely spurious due to failure to control for these factors. We also present results that the method developed by Rosenberg et al. (2022) is highly sensitive to noise in the dihedral bond angle distributions.

## Results

To test the robustness of the conclusions of Rosenberg et al. (2022), we re-create their analysis using four simulated datasets: (i) synonymous codon usage is completely random (i.e. the probability of seeing a codon for an amino acid with $n_{a}$ synonymous codons is $1/n_{a}$, the Uniform model), (ii) codon usage is determined by mutation biases (the Mutation-Empirical and the Mutation-ROC model) and (iii) synonymous codon usage is at the SMDE model. A key point of these simulations is that they do not consider position-specific information. The locations of codons should be (mostly) randomized in both simulated datasets, eliminating any or most of the signal related to dihedral bond angles, should it exist. We emphasize the point of the Uniform model is not to test if there is a signal of selection on codon usage related to bond angles, but to determine the extent to which the approach by Rosenberg et al. (2022) is prone to attributing noise to signal.

The computational pipeline and the publicly available data used to recreate their analysis were downloaded from the sources specified in Rosenberg et al. (2022). This pipeline was applied to all simulated coding sequences using the default settings. To analyze the simulated protein-coding sequences with the Rosenberg et al. (2022) pipeline, the represented protein-coding sequences were replaced with their simulated counterparts, retaining all other information from the real data, including the bond angles. Throughout the article, we will refer to “synonymous codon pairs” or “a pair of synonymous codons,” as the Rosenberg et al. (2022) pipeline compares the dihedral bond angle distribution of all possible combinations of synonymous codons.

### The Method of Rosenberg et al. (2022) is Prone to Attributing Noise to Signal

With codon usage simulated under the Uniform model, one would expect no signal relating codon usage to dihedral bond angles. However, 78 synonymous codon pairs were found to be significantly different in *β*-sheets in the randomly simulated protein-coding sequences compared to 58 in the real sequences. No synonymous codon pairs were significantly different for *α*-helices in either dataset.

**Fig. 1.**
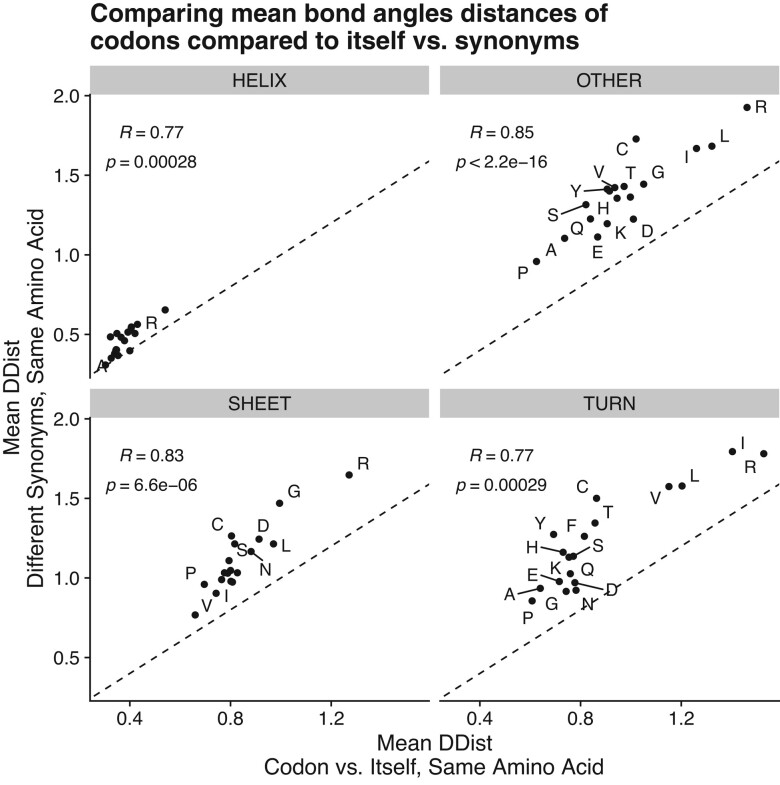
Comparison of the mean distances between bond angle distributions (the test statistic of Rosenberg et al. 2022) when a codon is compared to itself vs. its synonyms for each amino acid and secondary structure. These are based on the analysis of the real protein-coding sequences. Note that the distances in bond angle distributions when comparing a codon with itself represent noise, as the underlying distributions are the same.

The striking number of statistically significant results in *β*-sheets under completely random codon usage suggests the method developed by Rosenberg et al. (2022) conflates noise in dihedral bond angles with signal, potentially due to over-fitting of the kernel density to the bond angle distributions. As an additional control in their analysis, Rosenberg et al. (2022) applied their statistical test to compare the bond angle distributions of each codon to itself. As expected, the bond angle distribution of a codon is never significantly different from itself. However, there are still significant variations in the calculated test statistics (the distances between the bond angle distributions) for each codon compared to itself. This reflects the degree of random noise in the bond angle distributions for a given codon, as these distributions are not truly different. We tested if amino acids that tended to show greater distances between bond angle distributions within codons also showed greater distances between synonymous codons within each secondary structure group. We observed a strong correlation between the mean test statistics within codons and between codons when using the real protein-coding sequences in all four classes of protein secondary structure (Fig. 1). Perhaps more telling, we found that these correlations also exist in the analysis of the simulated data with completely random codon usage, potentially explaining why significant results were found where none should exist (supplementary fig. S1, Supplementary Material online). The correlations observed here suggest the Rosenberg et al. (2022) method is prone to detecting random noise.

### The Results of Rosenberg et al. (2022) are Mostly Consistent with the Selection-Mutation-drift Equilibrium Model of Codon Usage Bias

Although far from a complete model of the evolutionary processes shaping codon usage bias within a genome, the assumption of SMDE has proven to be a powerful model for understanding the factors shaping codon usage bias (Bulmer 1991; Harrison and Charlesworth 2011; Shah and Gilchrist 2011). We used ROC-SEMPPR to generate simulated protein-coding sequences under the SMDE model (Gilchrist et al. 2015). ROC-SEMPPR assumes codon usage is at SMDE and can estimate parameters reflecting natural selection on synonymous codon usage and mutation biases by accounting for variation in gene expression across protein-coding sequences. ROC-SEMPPR only considers how codon counts vary across protein-coding sequences and not within, making it blind to information relevant to position-specific effects, such as protein secondary structure and dihedral bond angles.

**Fig. 2.**
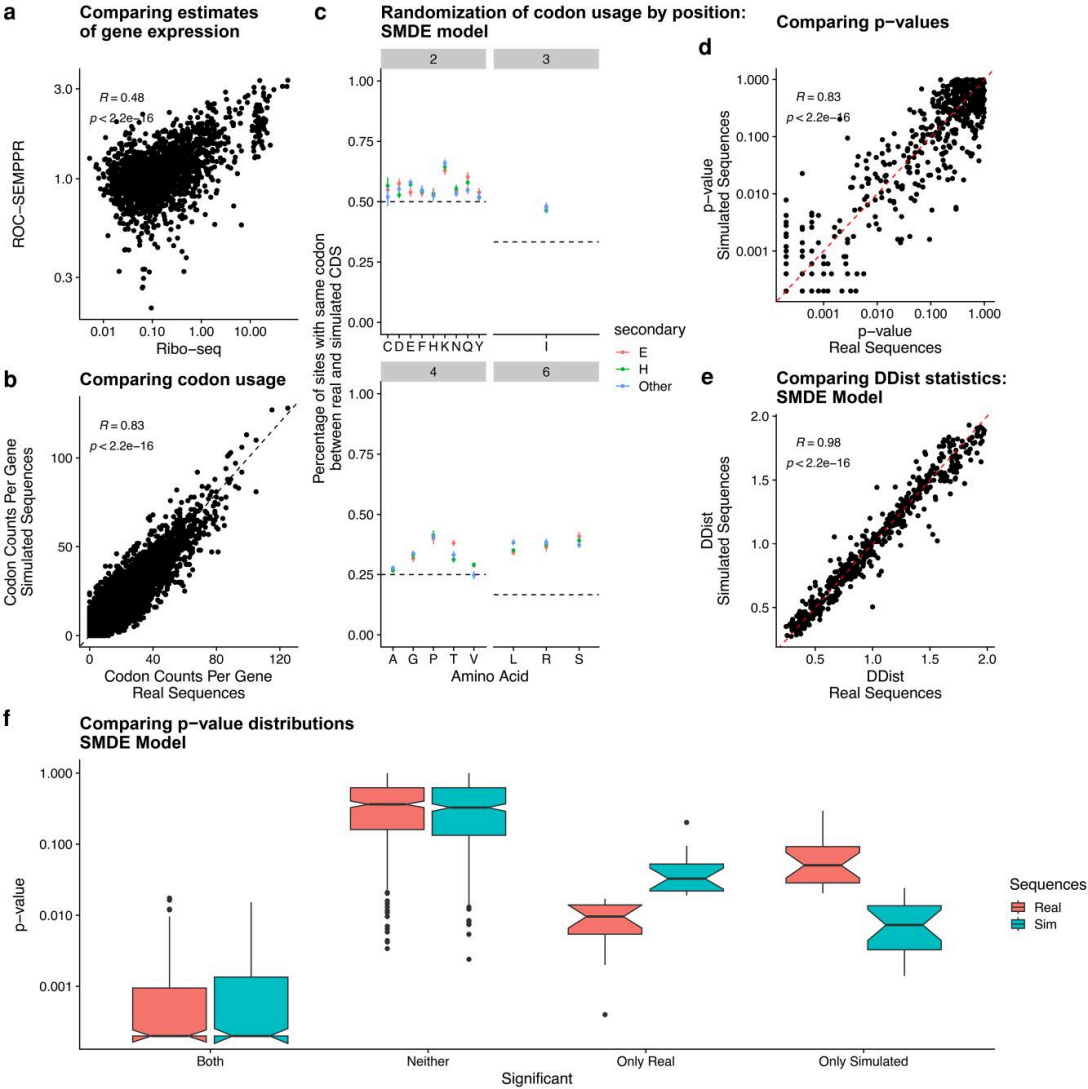
Codon usage simulated assuming SMDE generates correlations between synonymous codon usage and dihedral bond angles that are comparable to those observed using real data. a) Comparing estimates of ROC-SEMPPR predicted gene expression to Ribo-seq estimates taken from Mohammad et al. (2019). b) Comparing codon counts across all protein-coding sequences in the real and simulated data (ROC-SEMPPR SMDE). Each dot represents the number of occurrences of codon within a given gene. c) Percentage of amino acid sites that have the same codon at the same position in the same protein-coding sequence between the real and simulated data. The dashed line indicates the expectation under a completely random distribution. d) Comparison of *P*-values estimated from the Rosenberg *et al.* pipeline applied to the real and simulated data. e) Comparison of distribution distance statistics estimated from the Rosenberg *et al.* pipeline applied to the real and simulated data. f) Comparing *P*-value distributions for synonymous codons that were significant in both, neither, or only one of the real and simulated data analyses.

The *E. coli* K12 MG1655 protein-coding sequences were downloaded from NCBI-RefSeq (GCF_000005845.2). Rosenberg et al. (2022) did not restrict their analysis to a single strain of *E. coli*. Non-K12 MG1655 protein-coding sequences used by Rosenberg et al. (2022) were downloaded from the European Nucleotide Archive and appended to the *E. coli* K12 MG1655 protein-coding sequence FASTA file. We note that one protein-coding sequence (ENA AAL21040.1) used by Rosenberg et al. (2022) was annotated in the ENA as a *S. enterica* gene, but this was included for completeness. Excluding positions with missing codons in the real data, the simulated data contained 99% of the amino acid sites included in the real data. ROC-SEMPPR was fit to these protein-coding sequences using the AnaCoDa R package (Landerer et al. 2018) to estimate parameters reflecting natural selection on codon usage Δη, mutation bias ΔM, and an evolutionary average estimate gene expression *ϕ*. We note that the ROC-SEMPPR gene expression estimates were well correlated with estimates taken from Ribo-seq data (Mohammad et al. 2019) (Fig. 2A, Spearman rank correlation R=0.48), suggesting an overall good model fit.

Parameters estimated by ROC-SEMPPR were then used to simulate the codon usage of *E. coli* protein-coding sequences under the Mutation-ROC and SMDE models. Briefly, for each occurrence of an amino acid in a protein-coding sequence *g*, a synonymous codon was randomly sampled according to a multinomial distribution with the probability $p_{i,g}$ of observing synonymous codon *i* (of *N* synonymous codons) determined by the expression level of the gene $\phi_{g}$ and the natural selection Δη and mutation bias ΔM parameters estimated by ROC-SEMPPR for the relevant set of synonymous codons (see Equation (1) and see Gilchrist et al. 2015 for details).


(1) $$p_{i,g}=\frac{e^{-\Delta M_{i}-\Delta \eta_{i}\phi_{g}}}{\sum_{\;j=1}^{N}e^{-\Delta M_{j}-\Delta \eta_{j}\phi_{g}}}$$

In the case of the Mutation-ROC model, the selection term Δη is 0 for all synonyms, resulting in codon usage being determined solely by mutation bias. The Mutation-Empirical model is the same as the Mutation-ROC model, with mutation biases replaced with empirical estimates calculated from mutation rates obtained from previous mutation accumulation experiments in *E. coli* (Long 2018). A key difference between the Mutation-ROC and Mutation-Empirical models is that ROC-SEMPPR allows for variation in mutation biases across amino acids, which may account for potential context-dependent effects. As expected, the real and SMDE simulated protein-coding sequences had similar codon counts per gene (Fig. 2B, Spearman rank correlation R=0.83, p<2.2e−16). A weaker positive correlation was observed when codon counts were simulated under the Mutation-ROC model (Spearman rank correlation R=0.56, p<2.2e−16) and the Mutation-Empirical model (Spearman rank correlation R=0.64, p<2.2e−16), consistent with mutation bias being the dominant driver of codon usage bias across a genome (supplementary fig. S2, Supplementary Material online).

Although codon usage within the SMDE model simulated protein-coding sequences is similar to the real sequences, the positions of synonymous codons within the sequences are effectively randomized (Fig. 2C). For example, for the 4-codon amino acid valine (V), the percentage of times the same codon occurred at the same position within a protein-coding sequence between the real and simulated protein-coding sequences is approximately 25%. For many amino acids, there is a small upward bias relative to the uniform expectation (Fig. 2C, dashed lines). This is likely because some protein-coding sequences will be strongly biased towards certain synonymous codons and some codons are rarely used, such as the isoleucine codon ATA and arginine codons AGG and AGA. We observed similar results with protein-coding sequences simulated under the Mutation-ROC and Mutation-Empirical models (supplementary fig. S3, Supplementary Material online). Given the overall lack of agreement between the real and simulated sequences on a position-specific basis, the simulated sequences should contain little, if any, of the correlation between synonymous codon usage and dihedral bond angles, provided there is a general relationship between the two.

Overall, we found striking agreement between our analysis of the real and the simulated sequences, regardless of the model used for simulating (Table 1). We provide comparisons of the analysis of the real sequences and the different simulated sequences in the Supplementary Material online (supplementary fig. S4–S6, Supplementary Material online). This suggests there are a large number of factors unrelated to codon usage that may be driving apparent differences in the bond angle distributions. That said, sequences simulated under the SMDE model generally best agreed with results from the real data. For example, although the test statistics for each synonymous codon pair were highly correlated with the test statistics from the real data across all four simulations, only the test statistics from the SMDE simulated sequences generally fall along the y=x line across the range of values (supplementary fig. S4–S6, Supplementary Material online). Additionally, these high correlations observed when using the Uniform and Mutation models may be due to general differences in the distributions of the test statistics across secondary structures (supplementary fig. S7, Supplementary Material online), as these correlations are much weaker when conditioning on secondary structure.

**Table 1.** Overall similarity across analyses of simulated protein-coding sequences to the analysis of the real protein-coding sequences

Metric	Uniform	Mutation-Empirical	Mutation-ROC	SMDE
test statistic correlation	0.91	0.91	0.92	0.98
*P*-value correlation	0.73	0.7	0.75	0.83
# Significant (Total)	250	214	196	174
# Significant (Real and Sim.)	167 (98%)	142 (84%)	136 (80%)	154 (91%)
# Significant (Only Sim.)	83	72	60	20

Test statistics and *P*-values were compared using the Spearman rank correlation coefficient. # Significant (Total): number of synonymous codon pairs found to have significantly different dihedral bond angle distributions in the simulated data. # Significant (Real and Sim.): number of synonymous codon pairs found to have significantly different dihedral bond angle distributions in both the real and simulated data. Percentages are relative to the total observed in the real data (170 significant synonymous codon pairs). # Significant (Only Sim.): number of synonymous codon pairs found to have significantly different dihedral bond angle distributions only in the simulated data.

Analysis of the SMDE model simulated sequences found that 91% of significant synonymous codon pairs in the real data were also significant in the simulated sequences (Table 1). In agreement with this, the *P*-values and the distances between the bond angle distributions (DDist, to use the column name from pipeline output) between the two analyses were highly correlated (Fig. 2D,E, Spearman rank correlation R=0.83, p<2.2e−16 and R=0.98, p<2.2e−16, respectively, and Table 1). In cases where bond angle distributions for synonymous codons were only significant in one of the two datasets, the same codons tended to have a lower, but not significant, *P*-value in the other dataset (Fig. 2F). Taken together, these results suggest the interplay between mutation bias and natural selection on codon usage, as well as the effects of gene expression on the strength of selection, are driving a significant portion of the results observed in the real data. In the context of our results, the correlation between the distance metric and differences in codon-specific elongation speed observed by Rosenberg et al. (2022) suggests they are detecting signals related to selection for translation efficiency. We note the frequency of different types of secondary structures and amino acid usage—both of which may impact the distribution of dihedral bond angles—are weakly (anti-)correlated with gene expression (see Supplementary material online: *Secondary structure frequencies and amino acid biases weakly vary with gene expression* and supplementary fig. S8, Supplementary Material online), which may partially contribute to some of the apparent patterns; however, given the evidence that the Rosenberg et al. (2022) pipeline is likely to attribute noise to signal, it is difficult to make sound conclusions about potential gene-specific factors that may cause biases in the dihedral bond angle distributions.

## Conclusion

Much like Rosenberg et al. (2022) could not definitively say if synonymous codon usage determined dihedral bond angles or vice versa, we cannot definitively say there is no relationship between synonymous codon usage and dihedral bond angles. Such a relationship necessitates that the effects at the A-site of the ribosome are carried over through the ribosome tunnel (approximately 35–40 amino acids long) and any subsequent co-translational or post-translational protein folding. Our results suggest the observed differences between bond angle distributions of synonymous codons were substantially impacted by the relationship between codon usage and gene expression. Even so, the approach developed by Rosenberg et al. (2022) appears to be highly prone to detecting random noise in bond angle distributions, such that even subtle differences in amino acid usage, overall protein structures, or other factors that correlate with gene expression could cause apparent differences in bond angle distributions. Thus, while we believe the specific results of Rosenberg et al. (2022) were mostly due to the relationship between codon usage and gene expression, the method described in Rosenberg et al. (2022) appears to be generally unreliable given the default parameters. The relationship between codon usage, protein structure, and the protein folding process remains an exciting area of research, particularly as advances in molecular and structural biology allow for more detailed investigations and experiments. We note there are 16 synonymous codon pairs (9 SHEET, 6 TURN, 1 OTHER) in the real sequences that were not significant in our SMDE simulations. Although we suspect this is due to the imperfect nature of our simulations which cannot possibly account for all potential sources of variation in codon usage within and across genes, we cannot completely discount the possibility that there is a true mechanistic or causal relationship between codon usage and the resulting dihedral bond angle in some rare cases. Further empirical work is needed to ascertain if a mechanistic relationship between protein bond angles and codon usage exists. Importantly, this work further underscores the importance of controlling for gene expression – which modulates the relative impact of mutation biases and natural selection for translation efficiency in shaping codon usage across genes—when testing for correlations between codon usage and other molecular features (Cope et al. 2018; Cope and Gilchrist 2022).

## Supplementary Material

evae080_Supplementary_Data

## Data Availability

Relevant data and scripts can be found at https://github.com/acope3/Codon_usage_prot_structure_angles or through the relevant sources found in Rosenberg et al. (2022).
