# Supplementary material for: Re-examining Correlations Between Synonymous Codon Usage and Protein Bond Angles in Escherichia coli
Source: Genome Biol Evol. 2024 Apr 15;16(5):evae080. doi: 10.1093/gbe/evae080 (PMC11077309; doi:10.1093/gbe/evae080)
Supplement: evae080_Supplementary_Data [file evae080_supplementary_data.pdf]

Supplementary Material

Secondary structure frequencies and amino acid biases weakly vary with gene expression

What potential gene-specific biases could lead to significant differences in dihedral bond angle distributions between synonymous codons? A key result from Rosenberg *et al.* (2022) was that differences in bond angle distributions were present in β-sheets and not α-helices. We observe a weak correlation between the frequency of β-sheets in a gene and gene expression (supplementary fig. S8(right), Spearman rank correlation $R = -0.14$, $p = 0.002$). The correlation between gene expression and the frequency of α-helices in a gene is not statistically significant (supplementary fig. S8(left), Spearman rank correlation $R = 0.078$, $p = 0.085$). Amino acid usage is also known to correlate with gene expression in *E. coli* (Akashi and Gojobori, 2002). Based on a correspondence analysis of amino acid usage across genes, we observe that gene expression is also weakly correlated with the second, third, and fourth principal components (Spearman rank correlation $|R| < 0.21, p < 0.006$ in all cases), which in total "explain" 27.9% of the variation in amino acid usage across genes. The weak correlations of amino acid usage and secondary structure with gene expression may partially contribute to the apparent differences in the bond angle distributions between synonymous codons by introducing biases or further confounding comparisons between synonymous codons.

Supplementary Figures

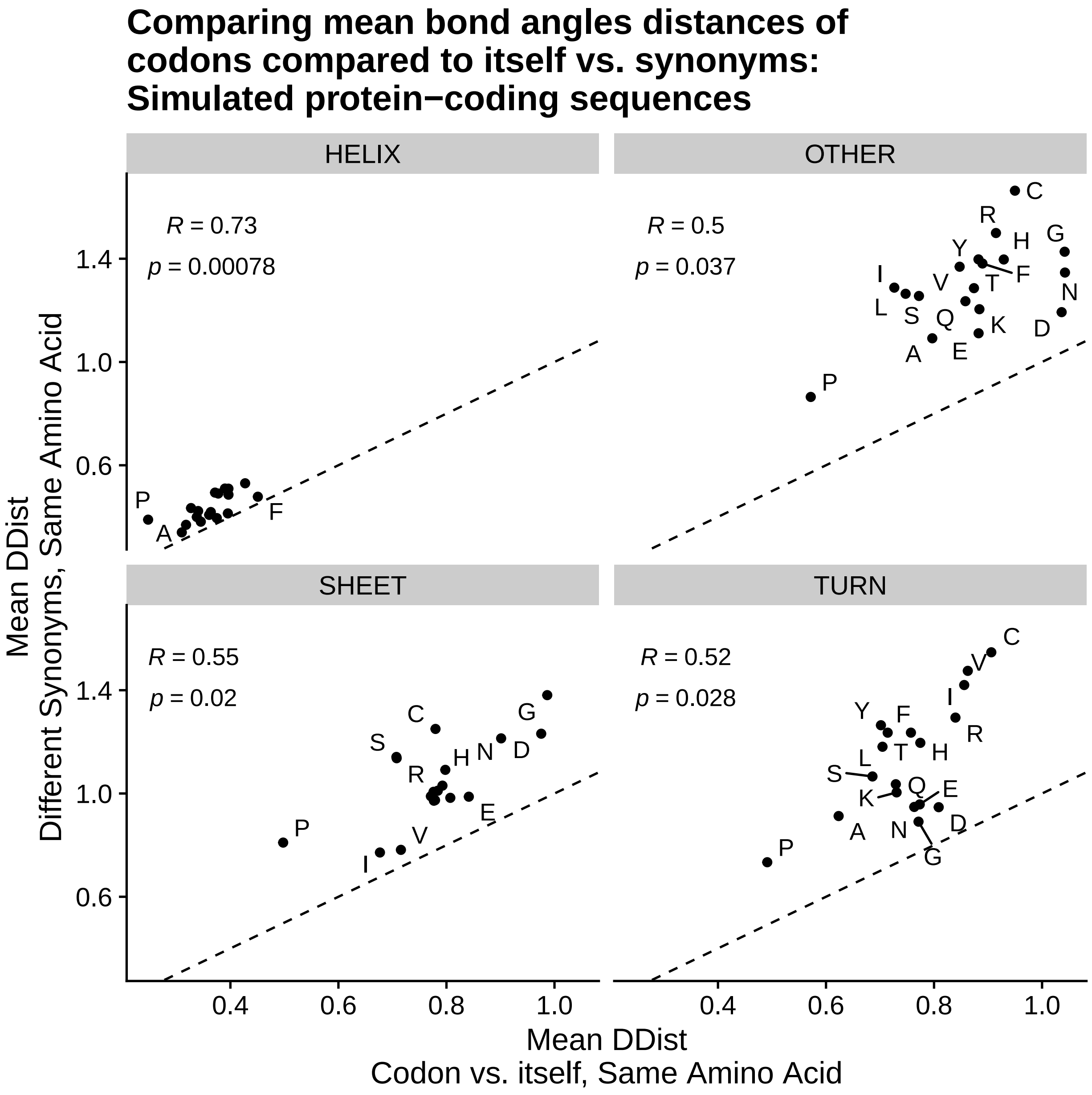

Figure S1: Comparison of the mean distances between bond angle distributions (the test statistic of Rosenberg et al) when a codon is compared to itself vs. its synonyms for each amino acid and secondary structure. These are based on the analysis of the randomly simulated protein-coding sequences. Note that the distances in bond angle distributions when comparing a codon with itself represent noise, as the underlying distributions are actually the same.

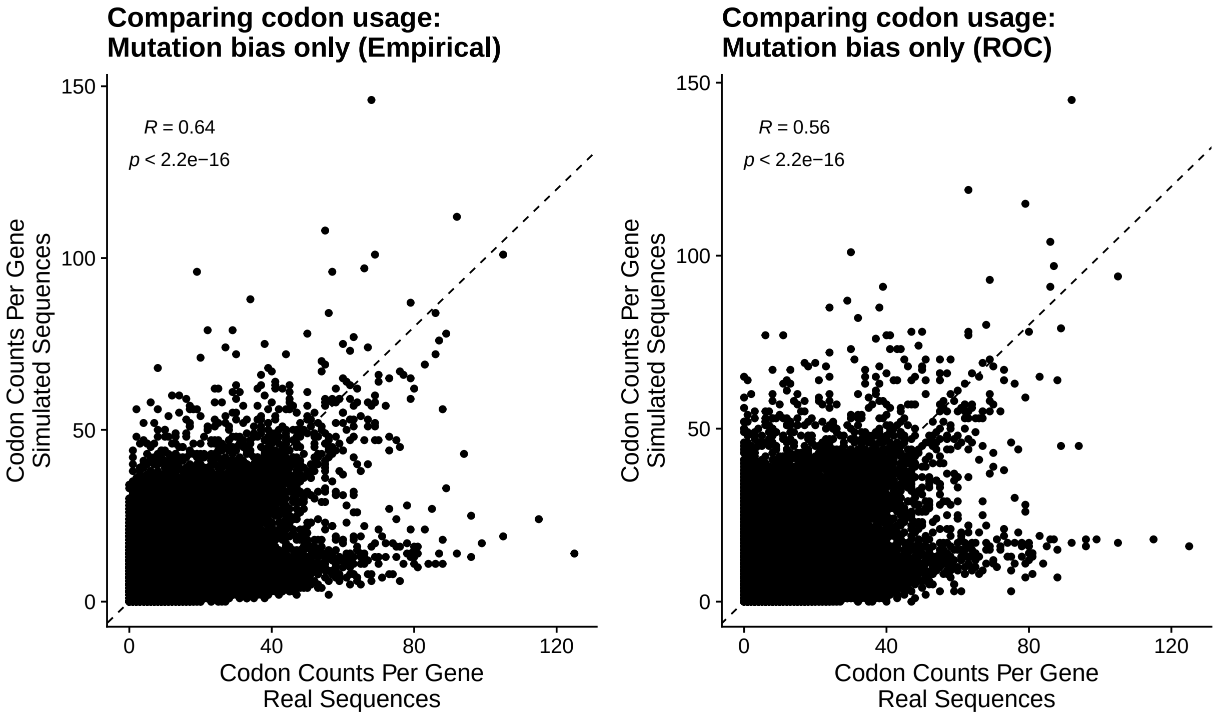

Figure S2: Comparing codon counts across all protein-coding sequences in the real and simulated data assuming codon usage is only shaped by mutation bias. Each dot represents the number of occurrences of codon within a given gene. Spearman rank correlation coefficient R and associated p-value are included on each scatter plot. (A) Simulation based on empirical mutation biases. (B) Simulation based on ROC-SEMPPR estimated mutation biases.

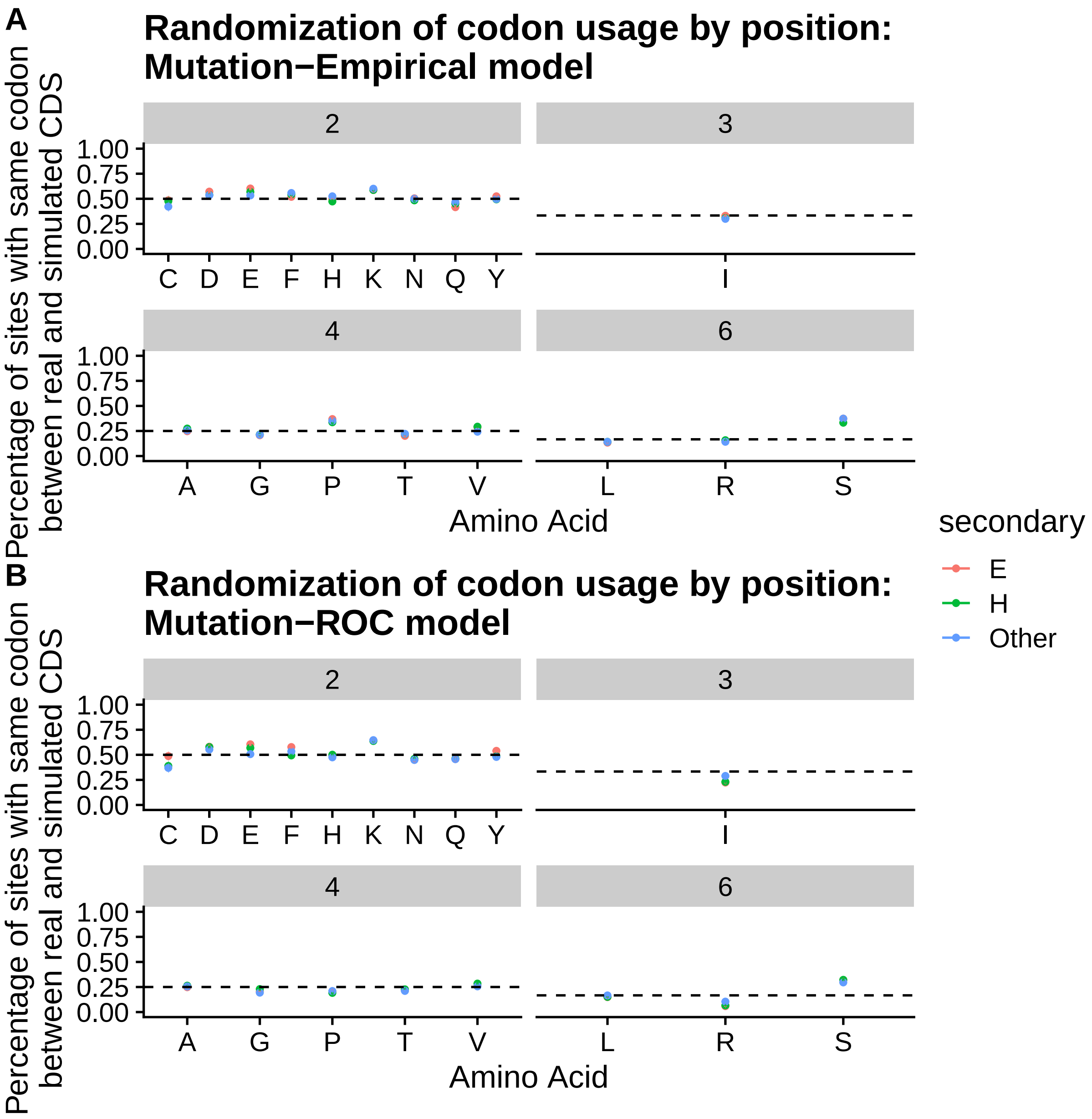

Figure S3: Percentage of amino acid sites that have the same codon at the same position in the same protein-coding sequence between the real and simulated data. The dashed line indicates the expectation under a completely random distribution. (A) Simulation based on empirical mutation biases. (B) Simulation-based on ROC-SEMPPR estimated mutation biases.

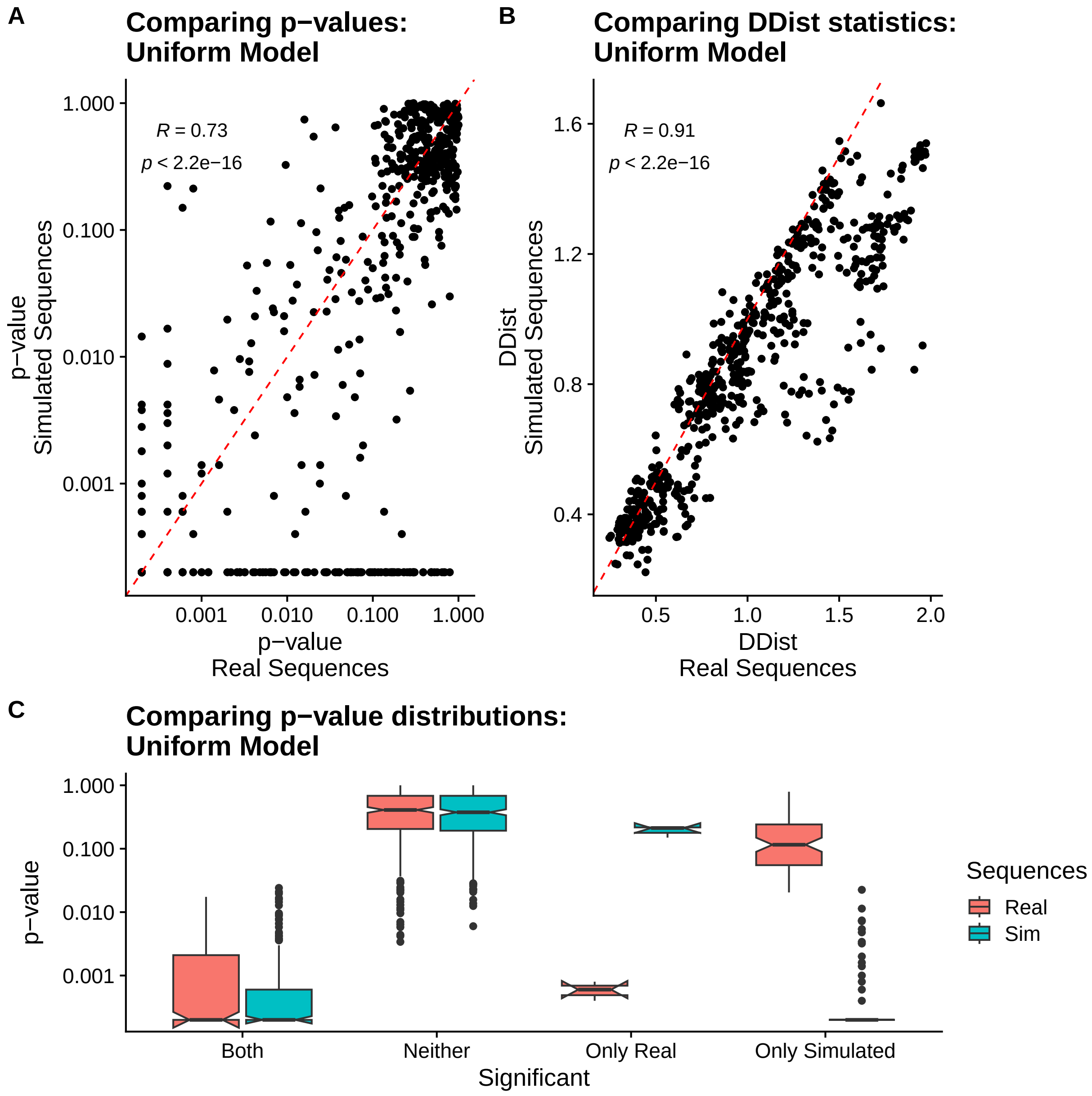

Figure S4: Comparison of results using the Rosenberg *et al.* (2022) method applied to real protein-coding sequences and sequences simulated via the Uniform model. (A) Comparing p-values. (B) Comparing the test statistics DDist. (C) Comparing p-value distributions for synonymous codons that were significant in both, neither, or only one of the real and simulated data analyses.

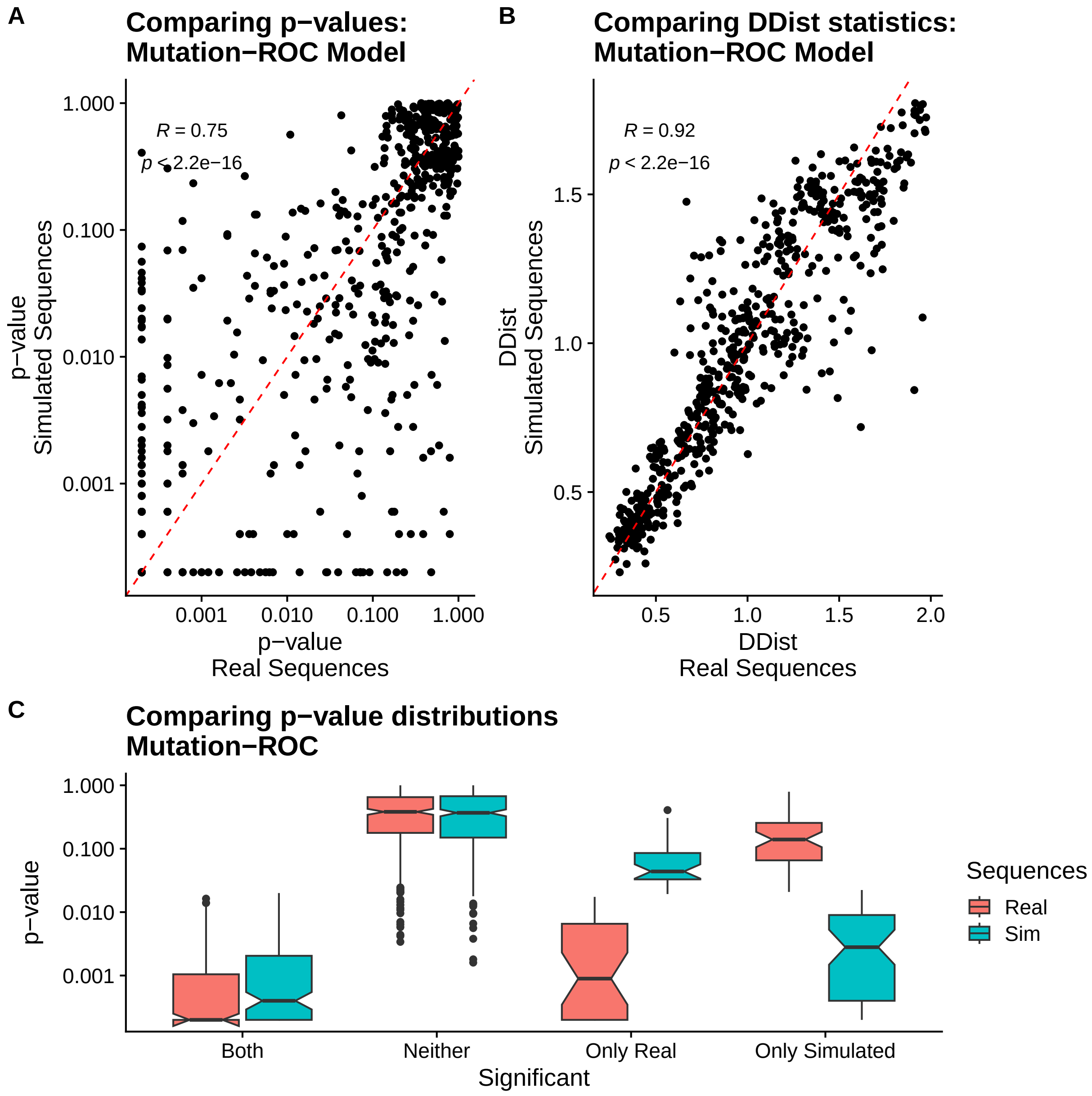

Figure S5: Comparison of results using the Rosenberg *et al.* (2022) method applied to real protein-coding sequences and sequences simulated via the Mutation-ROC model. (A) Comparing p-values. (B) Comparing the test statistics DDist. (C) Comparing p-value distributions for synonymous codons that were significant in both, neither, or only one of the real and simulated data analyses.

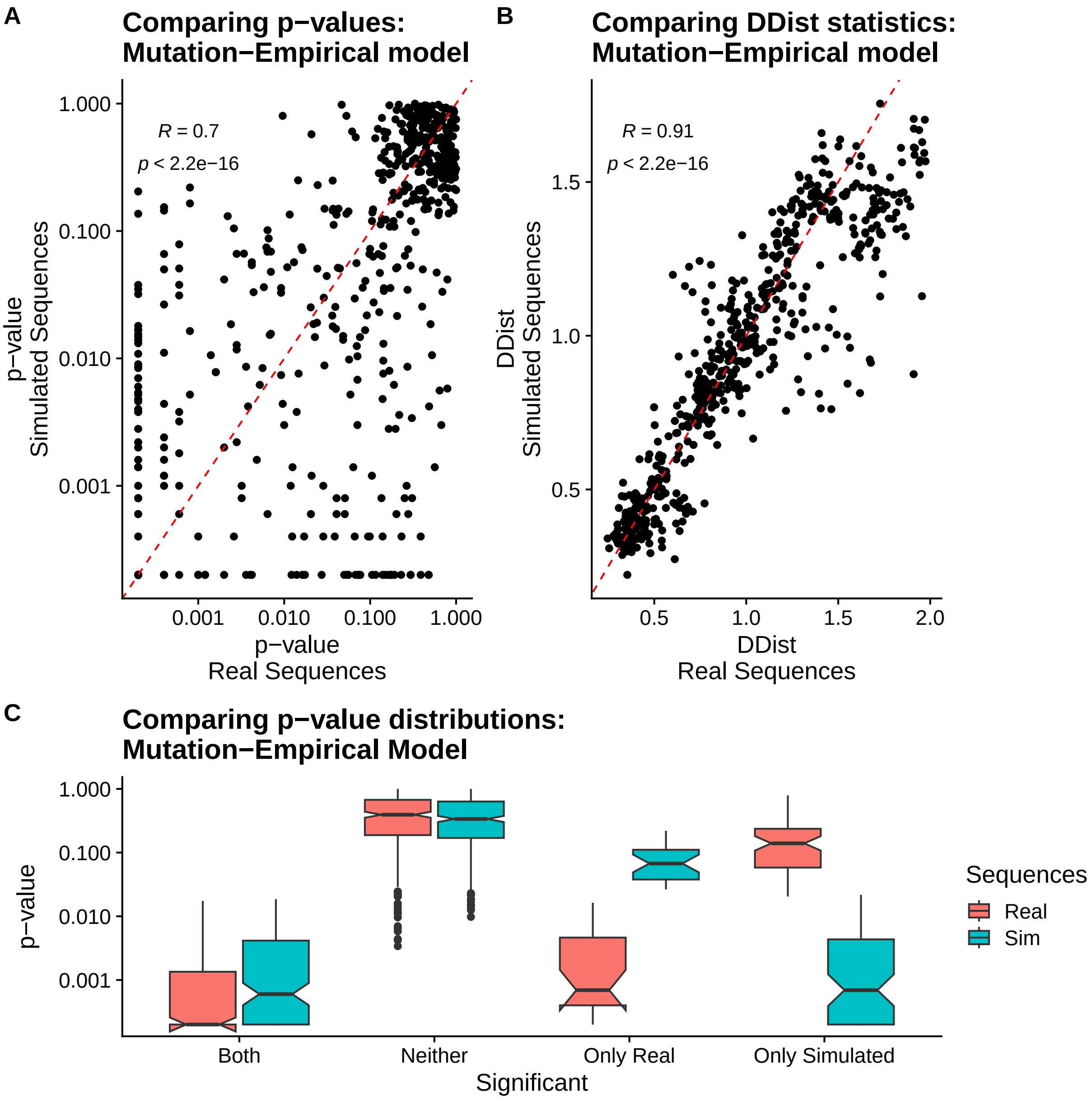

Figure S6: Comparison of results using the Rosenberg *et al.* (2022) method applied to real protein-coding sequences and sequences simulated via the Mutation-Empirical model. (A) Comparing p-values. (B) Comparing the test statistics DDist. (C) Comparing p-value distributions for synonymous codons that were significant in both, neither, or only one of the real and simulated data analyses.

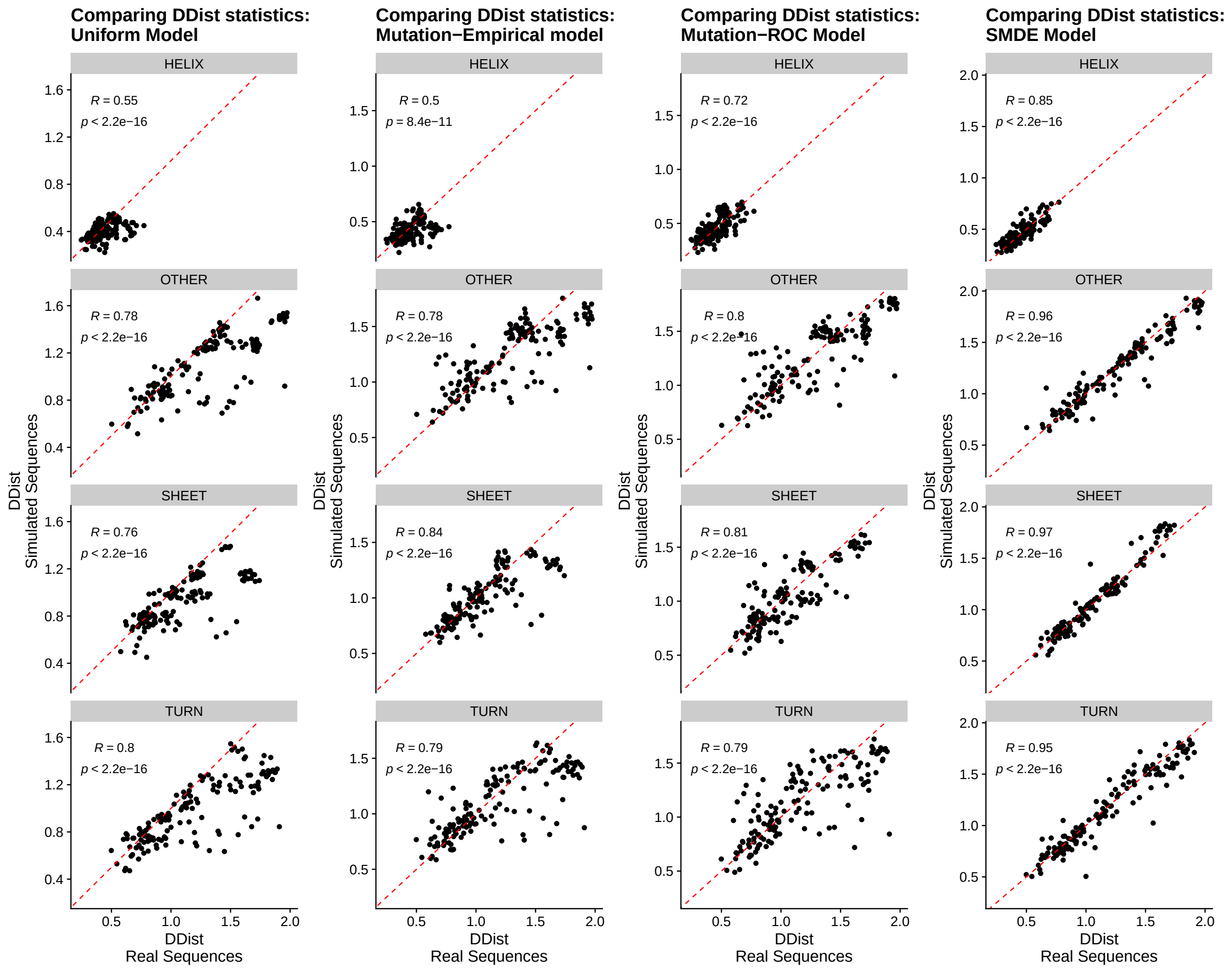

Figure S7: Comparison of test statistics (DDist) calculated using the Rosenberg *et al.* (2022) method based on the real protein-coding sequences vs. the 4 simulated datasets. Spearman rank correlation coefficients R and p-values are reported. The dashed red line indicates the $y = x$ line. When not separating the test statistics based on secondary structure, Spearman rank correlations between the real and simulated data are 0.91 (Real vs. Uniform), 0.91 (Real vs. Mutation-Empirical), 0.92 (Real vs. Mutation-ROC), and 0.98 (Real vs. SMDE).

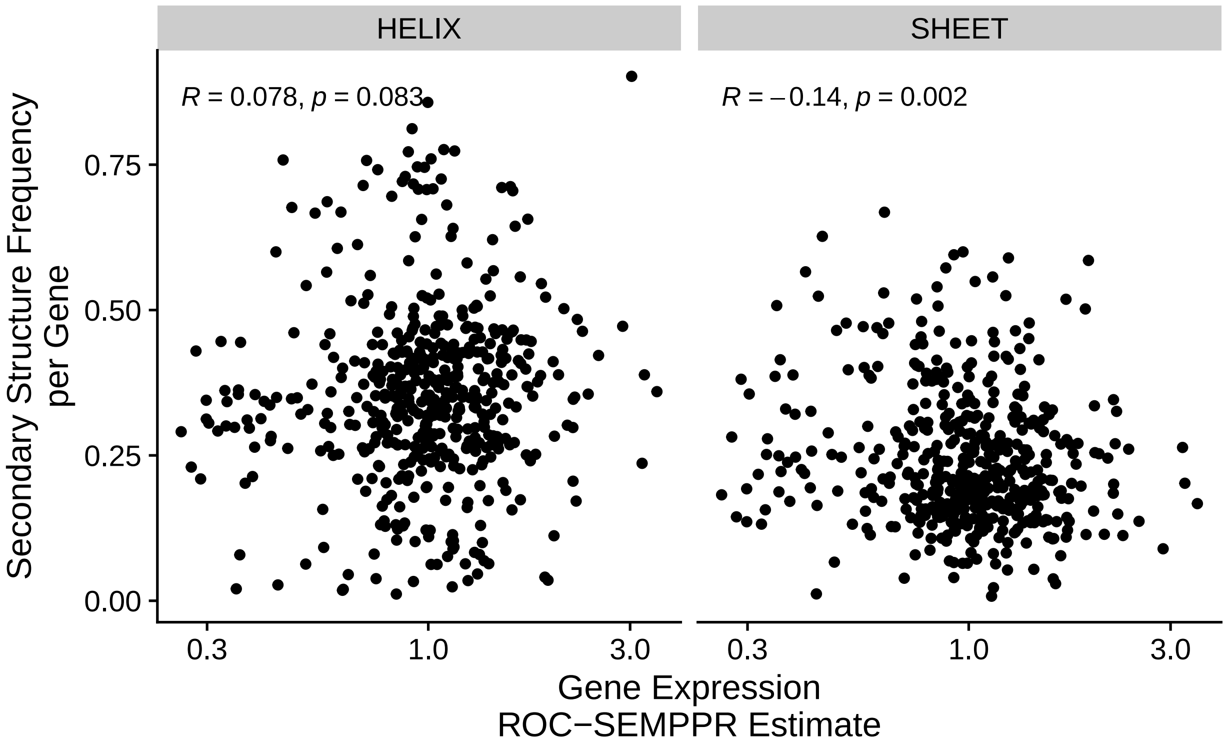

Figure S8: Correlation between gene expression estimate from ROC-SEMPPR ϕ and the frequency of each major secondary structure in each gene.

References

Akashi, H. and Gojobori, T. 2002. Metabolic efficiency and amino acid composition in the proteomes of escherichia coli and bacillus subtilis. *Proceedings of the National Academy of Sciences of the United States of America*, 99: 3695–700.

Rosenberg, A.A., Marx, A. and Bronstein, A.M. 2022. Codon-specific ramachandran plots show amino acid backbone conformation depends on identity of the translated codon. *Nature Communications 2022 13:1*, 13: 1–11.
